# Extrachromosomal Circular DNA in Cancer: Mechanisms and Clinical Applications

**DOI:** 10.1111/cpr.70040

**Published:** 2025-04-29

**Authors:** Jiajia Li, Peng Luo, Zhengrui Li, Qi Wang, Xufeng Huang, Keliang Wang, Ruo Wang, Runzhi Chen

**Affiliations:** ^1^ Hangzhou Geriatric Hospital, (Department of Stomatology), Affiliated Hangzhou First People's Hospital Chengbei Campus, School of Medicine, Westlake University Hangzhou Zhejiang China; ^2^ School of Rehabilitation Medicine, Binzhou Medical University Yantai Shandong China; ^3^ Department of Oncology Zhujiang Hospital, Southern Medical University Guangzhou Guangdong China; ^4^ School of Medicine, Shanghai Jiao Tong University Shanghai China; ^5^ Department of Gastroenterology Ningbo No. 2 Hospital Ningbo Zhejiang China; ^6^ Shengli Clinical Medical College of Fujian Medical University, Department of Breast Surgery, Fujian Provincial Hospital, Fuzhou University Affiliated Provincial Hospital Fuzhou China

**Keywords:** biomarkers, drug resistance, eccDNA, extrachromosomal circular DNA, therapeutic targets, tumour

## Abstract

Extrachromosomal circular DNA (eccDNA) has emerged as a critical area of cancer research due to its ubiquitous presence in tumour cells and significant role in tumorigenesis, progression and drug resistance. Recent studies demonstrate that eccDNA promotes cancer progression by influencing genomic instability, amplifying oncogenes, regulating gene expression and enhancing tumour cell adaptability to adverse conditions. While the precise mechanisms underlying eccDNA formation and its biological functions remain unclear, its potential applications in cancer diagnosis, prognosis and targeted therapy are gaining increasing recognition. This review summarises the latest advancements in eccDNA research, highlighting its potential as both a biomarker and a therapeutic target. Additionally, it emphasises the translational potential of eccDNA in clinical diagnostics and personalised treatment strategies, offering new perspectives for future cancer research and innovative therapies.

## Introduction

Extrachromosomal circular DNA (eccDNA) is a chromosome‐independent, circular, double‐stranded DNA molecule widely present in eukaryotic cells. Compared to traditional linear chromosomal DNA, eccDNA exhibits distinct biological characteristics, including highly heterogeneous and diverse structures. Its size ranges from a few tens of base pairs to millions, and its abundance and distribution vary significantly across different cell types and physiological states [1, 2]. Notably, eccDNA often carries complete genetic information, especially in tumour cells, where its high copy number and robust transcriptional activity underscore its critical role in tumorigenesis and progression [3].

In recent years, the biological functions of eccDNA have garnered increasing attention. Studies have demonstrated that eccDNA influences cell proliferation, differentiation and responses to environmental stressors by regulating gene expression [4]. In tumour cells, eccDNA enhances cellular viability and adaptability by amplifying and promoting the expression of tumour‐related genes. Additionally, eccDNA contributes to shaping the tumour microenvironment, promoting tumour heterogeneity and enhancing drug resistance. For instance, in oesophageal squamous cell carcinoma (ESCC), differential expression of eccDNA has been closely linked to tumorigenesis and progression, potentially influencing tumour behaviour through the regulation of specific signalling pathways [5].

**FIGURE 1:**
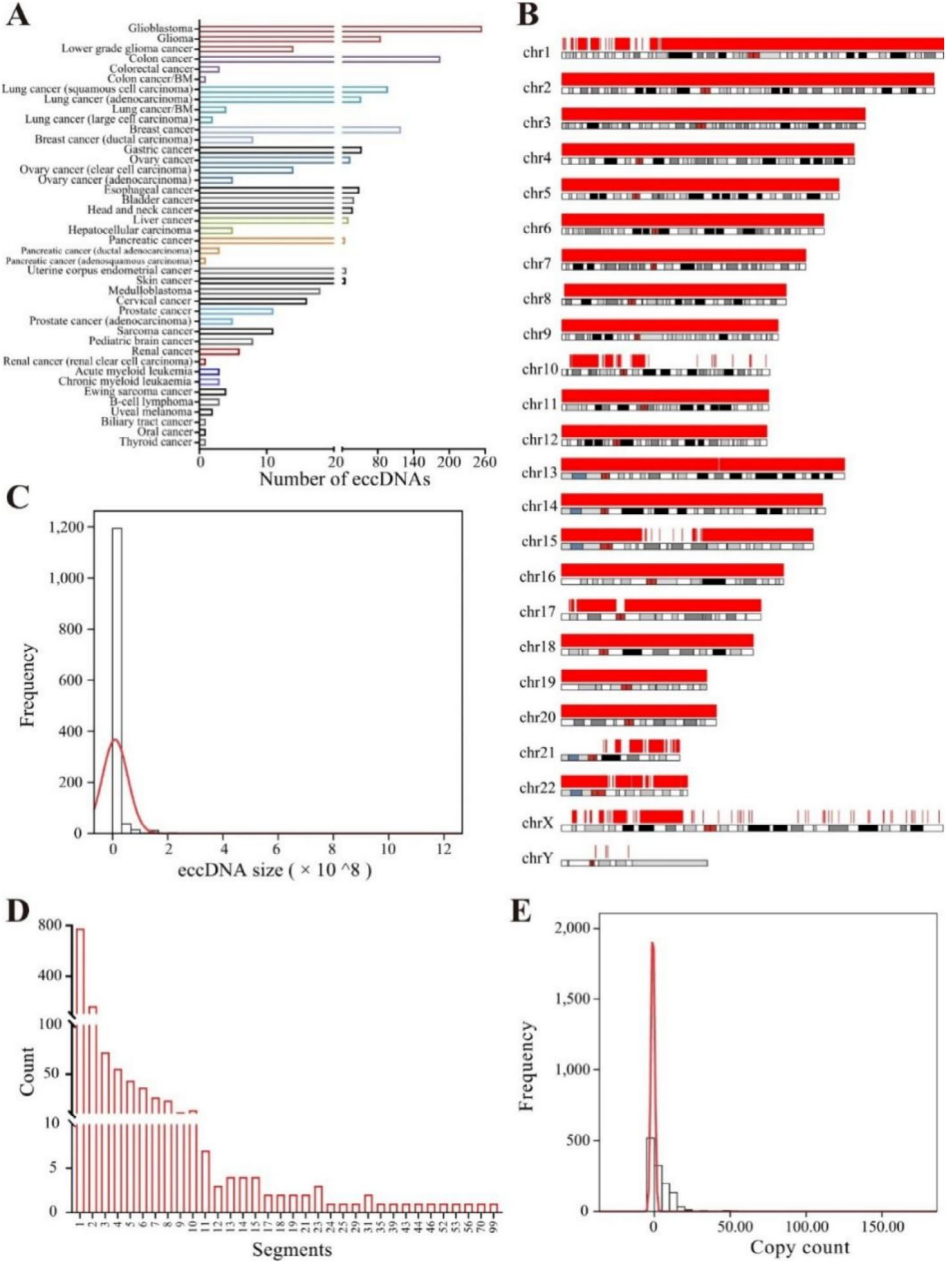
Statistics of eccDNAs in eccDNAdb [11]. (A) Distribution of eccDNAs in human cancers: This panel shows the number of eccDNAs identified in various human cancers, highlighting the differences in eccDNA prevalence across cancer types. Notably, glioblastoma, lower‐grade glioma and lung cancer subtypes show a higher abundance of eccDNAs, suggesting a potential role of eccDNAs in the pathogenesis of these cancers. (B) Distribution of eccDNA segments in chromosomes: The bar plots in panel B represent the distribution of eccDNA segments across different chromosomes. Chromosomes 1, 5 and 7 show a higher frequency of eccDNA segments, which could indicate specific regions of the genome more prone to eccDNA formation. (C) Distribution of eccDNA sizes: This panel illustrates the size distribution of eccDNAs, showing that the majority of eccDNAs are relatively small, with a peak at lower size values. The data suggest that smaller eccDNAs are more commonly found in the cancer samples analysed. (D) Distribution of eccDNA segment numbers: Panel D provides insight into the number of segments present within each eccDNA molecule. A significant portion of eccDNAs comprises fewer segments, though a smaller subset contains a higher number of segments, potentially correlating with more complex rearrangements or alterations in the genome. (E) Distribution of eccDNA copy counts: The final panel presents the distribution of eccDNA copy numbers. Most eccDNAs are found in low copy numbers, but a substantial fraction of the samples show higher copy counts, which may be indicative of eccDNA amplification events that could have diagnostic or prognostic implications.

With ongoing advancements in research, the pivotal role of eccDNA in tumour biology has become increasingly apparent. Its widespread presence in tumour cells and its contributions to genomic instability and tumour heterogeneity position eccDNA as a key focus for understanding tumour progression and drug resistance [6, 7, 8, 9, 10]. Furthermore, the rapid development of high‐throughput sequencing technologies has greatly facilitated the detection and characterisation of eccDNA (Figure 1), enabling researchers to explore its specific mechanisms in tumorigenesis and progression [12]. These advances not only enhance our understanding of tumour biology but also provide a novel theoretical foundation and technical framework for early tumour diagnosis and personalised therapeutic strategies.

**FIGURE 2:**
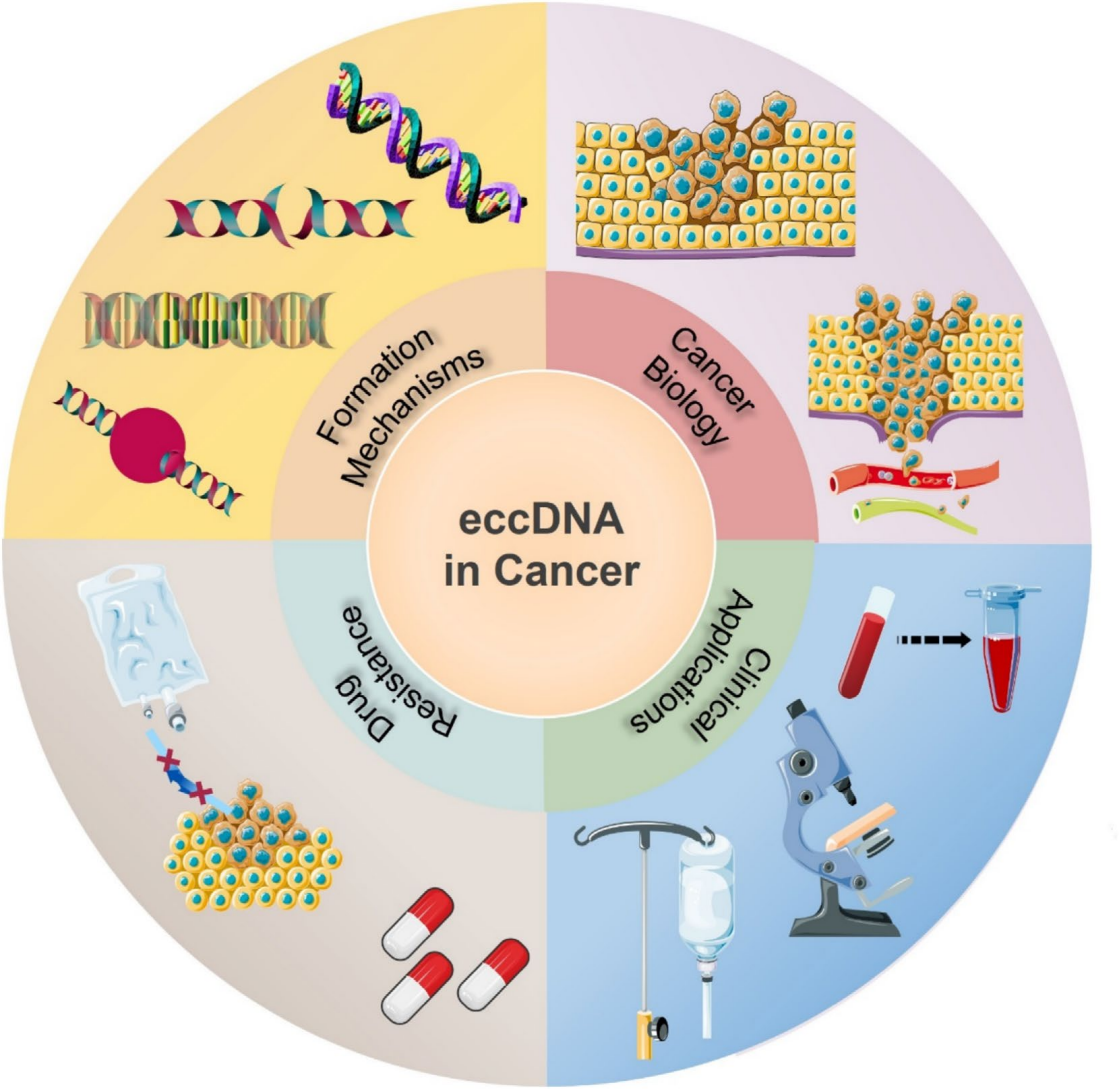
Schematic summary of the multifaceted roles played by eccDNA in cancer.

Overall in the present review, we comprehensively integrate the latest information on the mechanism of action of eccDNA in tumorigenesis, progression and drug resistance, and explore its application prospects in early diagnosis and precision treatment, proposing new insights into existing detection technologies and future research directions, thereby providing a new theoretical basis for future studies (Figure 2).

## Mechanisms Underlying eccDNA Formation

### Natural Resources of eccDNA

Abnormalities in DNA replication and transcription, together with dysregulation of DNA repair, are key drivers of eccDNA formation.

Research indicates that replication errors can lead to DNA breakage and recombination events, ultimately generating eccDNA13. It has been observed that in cancer cells, frequent gene amplification and transcriptional abnormalities significantly increase eccDNA formation, often resulting in eccDNAs that carry intact oncogenes and promote rapid tumour cell proliferation and metastasis.

On the other hand, RNA molecules produced during transcription may, under certain conditions, also be a source of eccDNA through reverse transcription, a phenomenon particularly evident in specific viral infections and tumour cells14.

Besides, dysfunctional DNA repair mechanisms represent a major driver of eccDNA formation. In normal cells, various DNA repair pathways, such as nucleotide excision repair and homologous recombination, ensure genome integrity. However, impaired DNA repair leads to strand breaks and abnormal recombination events, contributing to eccDNA formation. For instance, the breakage‐fusion‐bridge (BFB) cycle – often initiated by telomere loss or chromosomal breaks – involves repetitive cycles of chromosomal breakage, fusion, and bridging13. Furthermore, mutations or aberrant expression of DNA repair genes, such as BRCA1 and RAD51, have been strongly associated with excessive eccDNA accumulation, particularly in breast and ovarian cancers. Dysregulated repair mechanisms leading to eccDNA production not only exacerbate genomic instability but also provide tumour cells with adaptive advantages, including enhanced progression and drug resistance.

### Biosynthesis of eccDNA

The biosynthesis of eccDNA involves specific DNA repair pathways, particularly microhomology‐mediated end joining (MMEJ). MMEJ repairs DNA damage by utilising micro‐homologous sequences at DNA break ends, making it a key mechanism for eccDNA generation. Studies have shown that the presence of direct repeat sequences facilitates eccDNA formation, potentially through template slippage or non‐homologous end joining. Early investigations in plant systems suggested that eccDNA formation is a stochastic and universal process [13]. However, recent studies have illuminated the critical role of this mechanism in human diseases. For instance, in glioblastoma, eccDNAs carrying drug resistance genes have been found to form via the MMEJ pathway, significantly impacting tumour cell responses to treatment. Advances in high‐throughput sequencing technologies have further elucidated the mechanisms of eccDNA synthesis in various disease models, reinforcing its importance in genome remodelling and tumour progression. Investigating the biosynthetic pathways of eccDNA not only enhances our understanding of its cellular functions but also opens new avenues for developing therapeutic strategies targeting eccDNA.

## The Presence and Distribution of eccDNA in Tumours

### Characteristics of eccDNA in Different Types of Tumours

The distribution and biological roles of eccDNA vary across different tumour types, reflecting unique characteristics that have drawn significant attention in recent years. For instance, in colorectal cancer (CRC), eccDNA exhibits an aggregated distribution pattern, which correlates with the high expression of tumour‐associated genes. This suggests that eccDNA may contribute to tumorigenesis and progression by regulating key gene expression [14, 15]. In ESCC, the quantity and specific expression patterns of eccDNA positively correlate with tumour malignancy, with certain highly expressed eccDNAs being implicated in enhancing tumour invasiveness [16].

Similarly, in breast cancer, eccDNA is highly expressed in lymph node metastatic tissues, indicating a potential role in promoting tumour metastasis [17]. Recent studies have extended these findings to other tumour types. For example, in hepatocellular carcinoma (HCC), the elevated expression of specific eccDNAs has been associated with poorer patient prognosis and treatment resistance. In lung adenocarcinoma, eccDNA expression variations have been linked to alterations in tumour metabolic pathways, potentially influencing therapeutic outcomes. These findings highlight the significant heterogeneity in eccDNA distribution across tumour types, emphasising its potential as a diagnostic and prognostic marker. Future large‐scale studies and cross‐sectional comparisons of eccDNA across different tumour types will further elucidate its distribution patterns and clinical significance.

### Copy Number Variation of eccDNA and Tumour Heterogeneity

ecDNA contributes to tumour heterogeneity through its unique dynamic copy number variation (CNV) patterns. First, gene dosage effects mediated by ecDNA amplification create subpopulation‐specific oncogenic dependencies [7]. Single‐cell sequencing in HCC reveals that ecDNA carrying the c‐MYC oncogene exhibits clonal expansion patterns, with copy numbers varying from 5 to 32 per cell across subclones [18].

Second, the circular architecture enables stochastic segregation during mitosis. In EGFR‐mutant non‐small cell lung cancer, ecDNA harbouring the T790M resistance mutation is unequally distributed via micronuclei formation [19].

Third, chromatin remodelling on ecDNA creates privileged transcriptional hubs. Comparative ATAC‐seq analysis in CRC shows ecDNA loci exhibit 3.2‐fold higher chromatin accessibility than their chromosomal counterparts (ΔATAC score = 4.7, *q* < 0.01), particularly at ABCG2 drug resistance loci [20]. This epigenetic privilege allows simultaneous activation of multiple resistance pathways (WNT/β‐catenin and NOTCH) in single cells, a phenomenon undetectable in ecDNA‐negative tumours.

Moreover, CNV of eccDNA contributes to genomic differences among tumour cells, thereby exacerbating tumour heterogeneity. This heterogeneity enables tumour cells to adapt more effectively to therapeutic pressures, facilitating treatment resistance. The genomic remodelling capabilities of eccDNA provide a new perspective for developing personalised, eccDNA‐targeted therapies. Understanding the relationship between eccDNA CNVs and tumour phenotypes through further research could provide strong support for advancing precision medicine strategies.

### The Interaction Between eccDNA and Tumour Microenvironment

eccDNA exerts its effects not only within tumour cells but also on the tumour microenvironment, often by being released into the extracellular space. Acting as a signalling molecule, eccDNA can influence the behaviour of tumour‐associated cells, modulating immune responses and cell‐cell interactions [7]. In lung and pancreatic cancers, eccDNA has been shown to promote tumour stem cell‐like properties, thereby enhancing tumour invasiveness and metastasis [19].

Furthermore, eccDNA may contribute to the development of drug‐resistant tumour phenotypes by altering the cellular composition and metabolic profile of the tumour microenvironment. This interaction adds to the complexity of tumour treatment and highlights the multifaceted role of eccDNA in shaping the tumour microenvironment. Future research into the interaction mechanisms between eccDNA and the tumour microenvironment is essential for advancing our understanding of tumour progression. These studies will also pave the way for innovative therapeutic strategies targeting eccDNA, with the potential to address tumour heterogeneity and treatment resistance more effectively.

## The Role of eccDNA in Tumorigenesis and Development

### Promotion of Tumour Cell Proliferation and Growth

eccDNA plays a pivotal role in promoting the rapid proliferation and growth of tumour cells by carrying complete oncogene sequences, facilitating gene amplification and exhibiting high transcriptional activity. This phenomenon of gene amplification is particularly pronounced in cancer, where it significantly upregulates oncogene expression, driving malignant tumour progression. Additionally, eccDNA formation is closely linked to mechanisms such as DNA damage repair, the BFB cycle and chromosome fragmentation. These processes not only contribute to oncogene amplification but also increase genetic heterogeneity, which serves as a major factor in therapeutic resistance [1].

### Enhancement of Tumour Cell Metastasis and Microenvironment Regulation

eccDNA enhances the metastatic potential of tumour cells by modulating intercellular signalling and shaping the tumour microenvironment. For instance, eccDNA promotes tumour invasiveness and migration by regulating the expression of metastasis‐related genes [21]. Moreover, eccDNA is closely associated with the “stemness” of tumour cells, enabling their survival and proliferation in adverse conditions. In breast cancer and other tumour types, specific subtypes of eccDNA have been linked to lymph node metastasis, indicating its potential as a biomarker for predicting metastatic risk [22].

By influencing the tumour microenvironment, eccDNA contributes to tumour cell adaptation and progression. These findings highlight the importance of eccDNA not only in tumour metastasis but also in establishing favourable conditions for tumour survival and expansion.

### Role in Cancer Stem Cell Characteristics

Recent studies have uncovered the significant role of eccDNA in cancer stem cell characteristics, which are key drivers of tumorigenesis, metastasis and recurrence. eccDNA enhances the stem cell‐like properties of tumour cells by regulating critical signalling pathways and facilitating malignant transformation. For example, eccDNA may encode microRNAs (miRNAs) associated with stem cell characteristics, enabling tumour cells to gain enhanced adaptability and survival advantages by modulating proliferation and survival signalling pathways [10].

This discovery provides a novel perspective on the formation and maintenance of cancer stem cells and underscores the potential of eccDNA as a therapeutic target. Targeting eccDNA could disrupt the self‐renewal and survival mechanisms of cancer stem cells, offering a promising avenue for combating tumour recurrence and metastasis.

## eccDNA and Tumour Drug Resistance

### The Role of eccDNA in Chemotherapy Resistance

Chemoresistance arises through three primary eccDNA‐mediated mechanisms: (1) Amplification of drug efflux transporters (eccDNA‐harboured ABCB1) reducing intracellular drug accumulation [23, 24]; (2) Ectopic expression of oncogenic isoforms (e.g., EGFRvIII) through circular DNA‐driven alternative splicing [25]; (3) Dynamic remodelling of tumour–stroma crosstalk via vesicular eccDNA transfer, as evidenced by pancreatic cancer models showing CAF‐mediated gemcitabine resistance through TP53‐mutant eccDNA exchange.

In addition to gene amplification and transcriptional regulation, eccDNA plays a significant role in enhancing tumour cell tolerance to chemotherapy. For example, eccDNAs carrying drug resistance genes, including those encoding for drug efflux pumps, contribute to the overexpression of these pumps, leading to a reduction in intracellular drug concentrations. Moreover, eccDNA formation is often associated with genomic recombination and mutation, which can modify critical drug targets, thereby diminishing the effectiveness of chemotherapy. A deeper understanding of these mechanisms is essential for unravelling the molecular basis of drug resistance in tumours and for the design of novel therapeutic strategies aimed at overcoming this challenge. Recently, CRISPR‐Cas9 technology has shown potential in targeting eccDNA to overcome chemoresistance by disrupting drug resistance genes [23, 26], such as ABCB1, and modifying oncogenic isoforms like EGFRvIII. These advancements offer promise for clinical applications aimed at combating eccDNA‐mediated chemoresistance.

### Regulation of Drug Targets by eccDNA

eccDNA also contributes to drug resistance by regulating the expression and functionality of drug target genes. For example, eccDNA can amplify drug target‐related genes, increasing their expression levels or modifying their functions, thereby inducing resistance. In head and neck cancer, amplification of the RAB3B gene through eccDNA has been shown to activate the autophagy pathway, leading to resistance against cisplatin [27].

Furthermore, eccDNA regulates resistance mechanisms for other chemotherapeutic drugs such as paclitaxel and gemcitabine. In lung and pancreatic cancers, eccDNA carrying mutations in EGFR and KRAS enhances tumour resistance to apoptosis induced by these drugs by activating the PI3K‐AKT and MAPK signalling pathways [28]. Such modifications enable tumour cells to adapt to therapeutic pressures, resulting in a multidrug‐resistant phenotype.

### eccDNA and Microenvironment Shaping in Drug Resistance

In addition to intracellular gene regulation, eccDNA also shapes the tumour microenvironment, exacerbating therapeutic challenges. Some eccDNA molecules are released into the tumour microenvironment via extracellular vesicles, where they influence the behaviour of surrounding cells [29]. For instance, in breast cancer, eccDNA released into the microenvironment enhances resistance to paclitaxel by promoting immune escape and metabolic remodelling.

Moreover, eccDNA can regulate stromal and immune cells in the tumour microenvironment to support drug‐resistant phenotypes. These molecules activate immunosuppressive signalling pathways, reduce anti‐tumour immune responses and alter extracellular matrix components, promoting tumour invasion and metastasis. This complex interplay highlights the multifaceted role of eccDNA in drug resistance, emphasising its potential as a therapeutic target.

## Prospects for Clinical Applications

### Application of eccDNA in Disease Diagnosis

eccDNA has emerged as a promising biomarker in disease diagnosis due to its close association with disease progression, including cancer, chronic kidney disease and gestational diabetes. Elevated levels of extracellular eccDNA (ucf‐eccDNA) in urine have been proposed as a non‐invasive biomarker for the early detection of chronic kidney disease, with promising diagnostic potential [19]. Similarly, specific eccDNA patterns identified in early pregnancy can serve as predictive markers for gestational diabetes, allowing for accurate risk stratification and screening [30].

In the context of cancer diagnosis, eccDNA analysis via liquid biopsy has demonstrated great promise in enhancing diagnostic accuracy [29]. For instance, blood‐based eccDNA signatures have been shown to distinguish early‐stage HCC from normal tissues. Furthermore, sub‐type‐specific eccDNA analysis in breast cancer supports molecular typing and prognostic evaluation, enabling a more tailored therapeutic approach [7].

These findings underscore eccDNA's potential as a non‐invasive tool for early disease detection and real‐time monitoring.

Recent advancements in liquid biopsy techniques, particularly the detection of eccDNA, have significantly improved our ability to detect and monitor diseases at earlier stages. Various techniques, such as single‐cell sequencing and circulating DNA capture methods, have been employed to enhance the sensitivity and specificity of eccDNA detection. For example, single‐cell sequencing provides a detailed and high‐resolution view of the genetic heterogeneity of eccDNA, which can be particularly useful in identifying tumour‐specific eccDNA signatures. On the other hand, circulating DNA capture technologies allow for more efficient isolation of eccDNA from complex biological samples, thereby improving the sensitivity of detection. However, each method comes with its own set of advantages and limitations. Single‐cell sequencing, while highly sensitive, can be cost‐prohibitive and technically challenging. In contrast, circulating DNA capture methods are more cost‐effective but may be limited in their ability to detect low‐abundance eccDNA fragments. Together, these advancements highlight the growing potential of eccDNA as a tool for disease diagnosis, although further optimisation of detection technologies is still required to fully realise its clinical potential.

### The Potential of eccDNA in Tumour Therapy

The unique characteristics and functions of eccDNA in tumours make it an attractive therapeutic target. By promoting gene amplification and regulating gene expression, eccDNA contributes to genomic instability and drug resistance, providing a theoretical basis for therapeutic intervention. For instance, CRISPR‐Cas9 technology has been employed to specifically target tumour‐associated eccDNA, effectively suppressing tumour progression by disrupting key drug resistance genes [31]. This approach highlights the potential of eccDNA as a therapeutic target in overcoming cancer resistance mechanisms.

Dynamic monitoring of eccDNA levels can also help evaluate treatment responses in real time, optimising personalised therapeutic strategies, minimising side effects and improving outcomes. Additionally, eccDNA‐based combination therapies, such as pairing targeted inhibitors with radiotherapy, have shown promise in overcoming drug resistance in tumours. These findings indicate that eccDNA‐targeted therapies are likely to become a cornerstone of precision medicine in the future.

### Challenges and Future Directions

Despite the promising applications of eccDNA, several challenges remain for its clinical implementation.

*Incomplete Understanding of Formation Mechanisms:* The processes underlying eccDNA formation and function in different diseases remain poorly understood, necessitating further basic research [32].

*Limitations of Detection Technologies:* Current detection methods, such as next‐generation sequencing (NGS), face challenges in terms of sensitivity, specificity and cost, particularly for early diagnosis and the detection of low‐abundance eccDNA. For instance, while NGS can provide high‐throughput data, it may not always be sensitive enough to detect low‐copy eccDNA, and the high cost remains a significant barrier to widespread clinical use.

*Lack of Standardisation:* The clinical translation of eccDNA research is hindered by the absence of standardised detection protocols and insufficient validation in clinical settings.

To address these challenges, future research should focus on the following directions:

*Constructing a Large‐Scale eccDNA Database:* Establish a systematic database to integrate eccDNA characteristics and functions across various diseases, providing a foundation for clinical applications.

*Optimising Detection Technologies:* There is a need to improve the sensitivity and specificity of detection methods. Real‐time PCR offers a potential solution for dynamic monitoring of eccDNA levels with greater sensitivity than traditional sequencing methods. Additionally, advancements in single‐cell sequencing could provide valuable insights into the detection of low‐abundance eccDNA in heterogeneous samples.

*Standardising Research Protocols:* Develop uniform standards for eccDNA detection and analysis to ensure reproducibility and comparability of results.

*Promoting Clinical Validation:* Conduct large‐scale trials to evaluate the utility of eccDNA in disease diagnosis and treatment, bridging the gap between laboratory research and clinical practice.

## Conclusion

The present review highlights the multifaceted role of eccDNA in tumorigenesis, progression, and drug resistance, emphasising its potential as a biomarker and therapeutic target. eccDNA plays a critical role in regulating tumour gene expression, promoting genomic instability and enabling tumour adaptation to therapeutic pressures. Its unique functions in the tumour microenvironment, including immune regulation and drug resistance formation, underscore its value in cancer diagnosis and treatment.

In diagnostics, eccDNA's abundance, sequence characteristics and distribution patterns support its use in non‐invasive liquid biopsies for early tumour detection and classification. In therapy, eccDNA's role in drug resistance provides a new direction for developing targeted treatment strategies, with promising results from CRISPR‐based approaches.

Despite these advances, significant challenges remain in elucidating eccDNA's mechanisms of action, improving detection technologies and translating findings into clinical practice. Through technological innovation, standardisation, and multidisciplinary collaboration, future research will unlock the full potential of eccDNA. This will provide more precise and effective diagnostic and therapeutic options for cancer patients while opening entirely new directions for tumour research and treatment.

## Author Contributions

Jiajia Li, Peng Luo, Qi Wang, Ruo Wang and Xufeng Huang analysed the literature and wrote the first version manuscript. Zhengrui Li drafted the figures. Keliang Wang and Runzhi Chen conceived the idea. Keliang Wang and Runzhi Chen reviewed and revised the manuscript. All authors gave the final approval of the submitted version.

## Ethics Statement

The authors have nothing to report.

## Conflicts of Interest

The authors declare no conflicts of interest.

## Data Availability

Data sharing not applicable to this article as no datasets were generated or analysed during the current study.
